# Microbiota fingerprints lose individually identifying features over time

**DOI:** 10.1186/s40168-016-0209-7

**Published:** 2017-01-09

**Authors:** David Wilkins, Marcus H. Y. Leung, Patrick K. H. Lee

**Affiliations:** School of Energy and Environment, City University of Hong Kong, B5423-AC1, Tat Chee Avenue, Kowloon, Hong Kong, Special Administrative Region of China

**Keywords:** Built environment, Microbiota, Skin microbiota, Forensics

## Abstract

**Background:**

Humans host individually unique skin microbiota, suggesting that microbiota traces transferred from skin to surfaces could serve as forensic markers analogous to fingerprints. While it is known that individuals leave identifiable microbiota traces on surfaces, it is not clear for how long these traces persist. Moreover, as skin and surface microbiota change with time, even persistent traces may lose their forensic potential as they would cease to resemble the microbiota of the person who left them. We followed skin and surface microbiota within households for four seasons to determine whether accurate microbiota-based matching of individuals to their households could be achieved across long time delays.

**Results:**

While household surface microbiota traces could be matched to the correct occupant or occupants with 67% accuracy, accuracy decreased substantially when skin and surface samples were collected in different seasons, and particularly when surface samples were collected long after skin samples. Most OTUs persisted on skin or surfaces for less than one season, indicating that OTU loss was the major cause of decreased matching accuracy. OTUs that were more useful for individual identification persisted for less time and were less likely to be deposited from skin to surface, suggesting a trade-off between the longevity and identifying value of microbiota traces.

**Conclusions:**

While microbiota traces have potential forensic value, unlike fingerprints they are not static and may degrade in a way that preferentially erases features useful in identifying individuals.

**Electronic supplementary material:**

The online version of this article (doi:10.1186/s40168-016-0209-7) contains supplementary material, which is available to authorized users.

## Background

The transfer of personal skin microbiota traces to surfaces has been called a microbial ‘fingerprint’ [1, 2], reflecting both the individuality of skin microbiota [3] and the potential of such traces for use in forensic identification. Identification of individuals from their microbiota traces (hereafter ‘microbiota matching’) has been demonstrated with traces left on computer keyboards and mice [1], mobile phones [4], and household surfaces [5, 6]. However, an important forensic property of fingerprints is that they can persist unchanged on surfaces for long periods, allowing people to be reliably matched to fingerprints they deposited on a surface some time ago. Little attention has been given to the time for which identifiable microbiota traces persist on surfaces, and given that an individual’s skin microbiota can change significantly within weeks or months [7, 8], it is unclear whether an individual’s current skin microbiota would be similar enough to an older microbiota trace for a reliable identification to be made. As interest in the forensic potential of microbiota matching continues to grow, it is important that this practical limitation be investigated.

This study attempted microbial matching of individuals to their places of residence based on comparison of skin microbiota to household surface microbiota traces, to determine whether accurate microbial matching could be achieved even with large time delays between skin and surface sampling. While sources including outdoor air and pets [9, 10] can contribute to residential surface microbiota, occupant skin contributes a large proportion or majority of surface microbiota [5, 11, 12]. Occupants’ skin microbiota rapidly colonise a newly occupied residence, and a person leaving a residence can cause a decline in microbiota similarity within days [5], suggesting that household surface assemblages closely track changes in occupant skin microbiota. This study also examined Operational Taxonomic Unit (OTU) stability on skin and surfaces and applied survival analysis to determine whether the value of OTUs in identifying individuals was related to their temporal stability and chance of deposition from skin to surface.

## Results and discussion

**Fig. 1:**
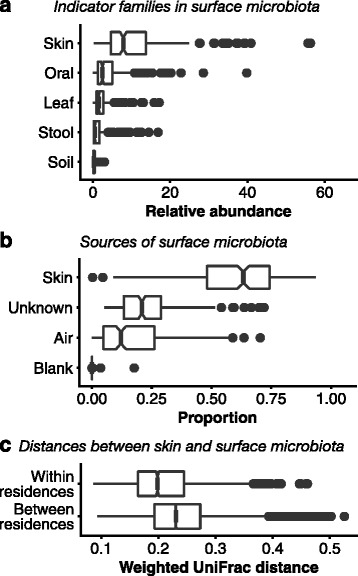
Summary of evidence that occupant skin is the major source of household surface microbiota. *Boxes* extend from first to third quartiles; *notches* indicate median and 95% CI (estimated as median $\pm 1.58\times \mathrm{I}\mathrm{Q}\mathrm{R}/\sqrt{n}$); *whiskers* indicate highest value within third/first quartiles $\pm 1.5\times \mathrm{I}\mathrm{Q}\mathrm{R}$; points indicate outliers. **a** Relative abundance in each surface sample of OTUs belonging to families identified by Dunn et al. [9] as indicative of human skin, human oral cavity, leaf, human stool and soil. **b** SourceTracker-estimated contribution of skin or air samples (same season) or negative control (blank) samples to microbiota in all household surface samples. **c** Weighted UniFrac distances between skin and surface samples (same season), showing distances between samples within the same residence or between different residences. A lower value indicates more similar microbiota

We collected microbiota samples from household surfaces, household air and residents’ skin in nine Hong Kong residences throughout 2014 (Table 1) and determined the microbiota compositions through 16S rRNA gene analysis. The taxonomic composition of surface samples confirmed that the majority of household surface microbiota originated from occupant skin. The most abundant family across all surface samples was *Moraxellaceae*, dominated by the skin - colonising genus *Acinetobacter*. Among the ten most abundant families were also the human skin-associated *Staphylococcaceae*, *Micrococcaceae*, *Corynebacteriaceae* and *Streptococcaceae*. However, there were also abundant populations of families likely derived from environmental sources such as soil and vegetation, including *Sphingomonadaceae*, *Methylobacteriaceae*, *Pseudomonadaceae*, *Rhodobacteraceae* and *Xanthomonadaceae*. We note that the 515F/806R primer set used in this study may underrepresent the phylum *Actinobacteria*, including the important human skin genus *Propionibacterium* [13]. Future investigations of microbiota matching may benefit from using additional or alternative primer sets better suited for human taxa. Using the list of indicator families for household microbiota sources developed by Dunn et al. [9], on average 10% (SE 0.52%) of surface microbiota abundance comprised OTUs from human skin-associated families, followed by 4.1% (SE 0.28%) for human oral cavity, 2.2% (SE 0.14%) for leaf, 1.6% (SE 0.15%) for human stool and 0.34% (SE $2.2\times {10}^{-2}$%) for soil (Fig. 1a; Kruskal-Wallis *p* < 0.05). We also used the Bayesian software tool SourceTracker [14] to estimate the proportional contributions of skin and household air to surface microbiota (Fig. 1b). On average, 60% (SE $1.1\times {10}^{-2}$%) of each surface sample’s microbiota was estimated to originate from occupant skin (within-season comparisons), compared to 17% (SE $8.8\times {10}^{-3}$%) from air, $7.6\times {10}^{-3}$% (SE $6.3\times {10}^{-4}$%) from negative control samples and 23% (SE $7.5\times {10}^{-3}$%) from unknown sources (Kruskal-Wallis *p* < 0.05). Finally, we examined whether an occupant’s skin microbiota resembled surface microbiota from their residence more than surface microbiota from other residences (Fig. 1c). On average, skin and surface samples from the same residence and season were more similar (mean weighted UniFrac [15] distance 0.21, SE $1.1\times {10}^{-3}$) than those from different residences in the same season (mean 0.24, SE $3.8\times {10}^{-4}$), a significant difference (Mann-Whitney *p* < 0.05). This confirms that household occupants in particular rather than human skin sources in general (via, for example, outdoor air, which in Hong Kong supports a large population of skin-associated phyla [16]) are the major source of household surface microbiota, as previously reported [5, 11, 17]. Skin samples from cohabiting individuals were also slightly but significantly more similar (mean weighted UniFrac distance 0.19, SE $1.2\times {10}^{-3}$, within-season comparisons, Mann-Whitney *p* < 0.05) than samples from non-cohabiting individuals (mean 0.23, SE $3.2\times {10}^{-4}$), suggesting some degree of microbiota exchange between individuals and/or via a common shared reservoir (e.g. household surfaces).

**Table 1:** Summary of collected samples

Sample property	Values (number of samples)
Type	Air (144), skin (380), surface (288)
Season	Winter 2014 (203), Spring 2014 (203), Summer 2014 (203), Autumn 2014 (203)
Residence	Admiralty A (68), Fortress Hill (68), Ma On Shan (108), Quarry Bay (88), Sai Wan (88), Sha Tin Wai (88), Tai Koo (108), Tuen Mun B (108), Wu Kai Sha (88)
Site	Bed headboard surface (36), bedroom air (36), blanket surface (36), forehead skin (76), fridge door seal surface (36), kitchen air (36), kitchen ventilator surface (36), left forearm skin (76), left palm skin (76), living room air (36), remote control surface (36), right forearm skin (76), right palm skin (76), shower curtain surface (36), toilet air (36), toilet flush button surface (36), TV screen surface (36)
Individual (skin only)	Admiralty A z (20), Fortress Hill z (20), Ma On Shan w (20), Ma On Shan x (20), Ma On Shan y (20), Quarry Bay y (20), Quarry Bay z (20), Sai Wan y (20), Sai Wan z (20), Sha Tin Wai x (20), Sha Tin Wai y (20), Tai Koo x (20), Tai Koo y (20), Tai Koo z (20), Tuen Mun B w (20), Tuen Mun B x (20), Tuen Mun B y (20), Wu Kai Sha w (20), Wu Kai Sha y (20)

**Fig. 2:**
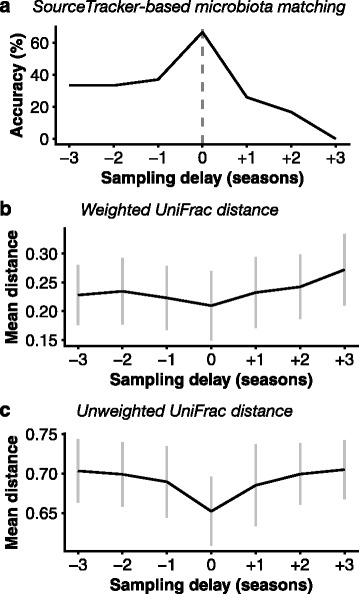
Effect of sampling delay on microbiota matching and UniFrac distance between skin and surface samples. Sampling delay is the number of seasons’ difference between the collection of skin and surface samples, with a positive delay indicating surface samples were collected after skin samples, while a negative delay indicates skin samples were collected after surface samples. **a** Accuracy of SourceTracker-based microbiota matching of skin to surface samples. Accuracy is determined as the proportion of residences for which SourceTracker estimated the correct set of occupants’ skin microbiota as the major source for the residences’ surface microbiota. **b** Weighted and **c** unweighted UniFrac distances between skin and surface samples from the same household. Samples with smaller distances have more similar community compositions. *Dark line* represents the mean of distances between all pairwise combinations of one skin sample and one surface sample from the same household. *Vertical grey lines* give the standard deviations

We used SourceTracker to perform microbiota matching of individual occupants (or, in the case of multi-occupant households, groups of occupants) to their households, as previously described [5, 6]. When skin and surface samples collected at the same time were used, the correct occupant or occupants were identified in 67% of cases (Fig. 2a), in keeping with the 58–77% accuracy rates previously reported for this method [5, 6]. The accuracy rate was highly sensitive to the number of individuals or groups of individuals to which a household could be matched. When microbiota matching was repeated against randomly selected subsets of potential matches, accuracy increased linearly as the size of the subset decreased, reaching 94% when there were only two possible matches (Additional file 1: Figure S1). This implies that in any practical forensic application of microbial matching, careful selection of the pool of potential matches may be one of the most important determinants of accuracy.

When we used surface samples collected a season or more after skin samples, the rate fell, with no accurate matches made after a delay of three seasons. Likewise, when we used skin samples collected after surface samples, the accuracy fell to 33% for delays of two or three seasons. While accuracy decreased with delay in both directions, skin-after-surface delays tended to yield a higher accuracy rate than surface-after-skin delays of the same magnitude. This effect of delay on matching is consistent with previous reports that the human skin microbiota changes with time [7, 8] and that microbiota traces deposited on surfaces begin to degrade within hours even in the absence of cleaning or other mechanical removal [18]. In this study, as the residences were continuously occupied, the most likely mechanisms for OTU loss are cleaning and other household activities as well as displacement of older traces by newly acquired skin OTUs. In other circumstances, such as the deposition of a trace in a public space or in a space vacated soon after the deposition, the degradation of identifying traces may occur faster in the absence of continuous deposition from the original host.

We confirmed these results by comparing UniFrac distances between skin and surface samples, another method that has been successfully employed for microbiota matching [1]. The mean distance between skin and surface samples from the same household was lowest for samples taken contemporaneously, and increased with increasing delay between skin and surface sampling in both directions (Fig. 2b, c), with the exception of a small decrease in the weighted UniFrac distance between skin-after-surface delays of two to three seasons. Weighted but not unweighted UniFrac distances were generally lower for skin-after-surface delays than surface-after-skin delays of the same length of time, similar to the skewed distributions of SourceTracker matching accuracy.

**Fig. 3:**
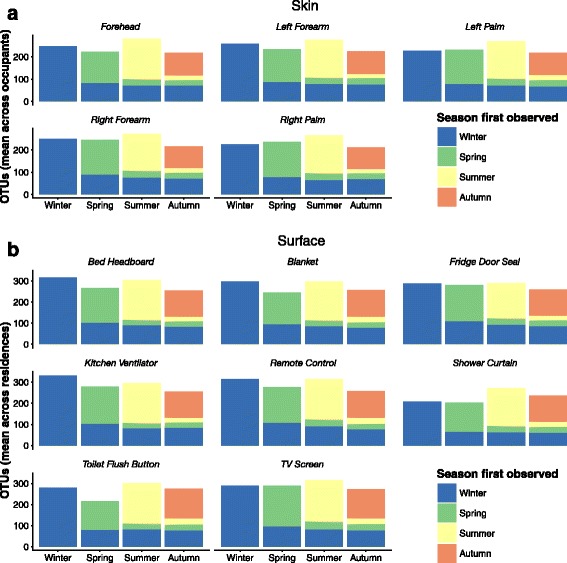
Seasonal OTU stability on **a** skin and **b** surface. Read counts have been normalised across samples. Counts represent means across **a** individuals or **b** residences

While this skew could be attributed to technical variation in sampling and/or sequencing, this would most likely produce random variation in accuracy rates and UniFrac distances rather than a systematic skew. Additionally, technical variation would not explain why the weighted but not unweighted UniFrac distances exhibit this skew. An alternative explanation is that OTUs tend to persist longer on skin than on surfaces. If OTUs were frequently exchanged between skin and surfaces, creating a shared OTU pool, but these OTUs persisted for longer on skin than on surfaces, over time skin microbiota would more resemble the past shared pool and therefore past samples of both types. To examine the persistence of OTUs on skin and surfaces, each OTU in each sample was mapped to the season in which it was first observed (Fig. 3). In both skin and surface samples, an average of 32% of OTUs in non-winter samples had been present in the sampled body site or household surface in winter, the season in which observations began. While each season after winter saw an influx of new OTUs (mean 58% of OTUs per sample), these same OTUs consistently declined to comprise an average of 19% per sample in the following season. This suggests that both skin and surface microbiota contained populations of stable OTUs that persisted for many seasons, comprising ~30% of the OTUs present at any time, as well as transient OTUs that persisted for one season or shorter. OTUs that persisted for more than one season comprised a slightly but significantly (Mann-Whitney *p* < 0.05) higher proportion by relative abundance of skin samples (mean 83% per sample) than surface (81%), while OTUs present in all four seasons comprised a mean abundance of 60% of skin samples but 57% of surface samples (Mann-Whitney *p* < 0.05). This may explain the skewed distributions of both microbiota matching accuracy and weighted UniFrac distances over different sampling delays, as the higher abundance of stable OTUs on skin could cause shared microbiota to persist for longer on skin than on surfaces. This would also account for the lack of skew in the distribution of unweighted UniFrac distances, as this distance varies with OTU diversity but not abundance.

We hypothesised that some OTUs are more useful for microbial matching than others, as they uniquely identify or are strongly associated with an individual or subset of individuals. However, by virtue of their specificity, these OTUs may be particularly susceptible to loss from skin microbiota [7], failure to be deposited on a surface as a microbiota trace, or loss from a deposited trace. To test whether this is the case, we used two measures of the identifying potential of OTUs, indicator value [19] and hitting set membership, and examined the relationship between these measures and OTU stability and deposition from skin to surface. Hitting sets [7], as applied to microbiota matching, are algorithmically determined minimal sets of OTUs that uniquely identify individuals within a cohort, thereby making membership of an OTU in a hitting set a useful marker of that OTU’s value in microbiota matching. Franzosa et al. reported that while hitting sets are relatively stable across time in gut and oral microbiota, allowing up to 80% accurate re-identification of individuals over time, skin microbiota OTU hitting sets are relatively unstable and do not permit accurate identification on repeat sampling [7]. We found a similar result in this dataset (Additional file 1: Figure S2), consistent with the hypothesis that the decreasing accuracy of microbiota matching over time is related to the loss of highly identifying OTUs. While the family *Moraxellaceae* was the most commonly represented in the hitting sets, as it was among all skin microbiota, overall the taxonomic distribution of hitting set OTUs did not closely resemble that of the total skin microbiota, with non-skin-associated families such as the *Sphingomonadaceae*, *Rhodobacteraceae* and *Weeksellaceae* among the most common hitting set members (Additional file 1: Table S1).

**Fig. 4:**
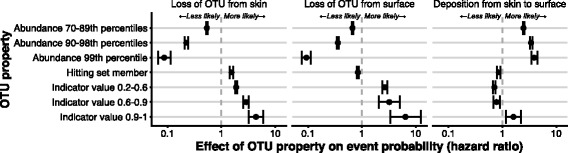
Effects of OTU properties on the probability of OTU loss and deposition events. OTU abundances were binned by rank percentile, with a higher percentile bin indicating a more abundant OTU, and indicator values by value, with a higher value indicating an OTU more specific for and faithful to an individual’s microbiota. OTUs with the ‘hitting set member’ property are those found to be most useful for uniquely identifying an individual and body site. *Error bars* represent 95% confidence intervals. A hazard ratio >1 indicates that the covariate increases the probability of the event relative to the baseline, while a value <1 indicates the covariate decreases the probability. No hazard ratios were calculated for the 0–69th abundance percentiles, indicator values between 0 and 0.2 or for non-membership of the hitting set, as the coefficients associated with these covariates are implicit by exclusion from the other covariate levels. The *x*-axis has been log_10_ scaled

We used Cox proportional hazard models to relate OTU abundance, indicator value and hitting set membership to the probability of an OTU being lost from either skin or surfaces, or of being deposited from skin to surface in a microbiota trace. Abundant OTUs were substantially less likely to be lost from either skin or surface (Fig. 4), with OTUs in the 99th abundance percentile <0.1× as likely to be lost relative to the baseline probability. OTUs with higher indicator values and OTUs that belonged to hitting sets were more likely to be lost from skin, with an indicator value >0.9 (high specificity and fidelity) associated with a 4.4× increase in the probability of being lost from a sample, and membership in a hitting set associated with a 1.5× increase. Given that the failure of hitting sets to successfully re-identify individuals over time is largely driven by false negatives (Additional file 1: Figure S2), this implies that the degradation of skin hitting set performance over time is driven mainly by loss of identifying OTUs, rather than by hitting set OTUs becoming more prevalent and thereby less useful for identification [7]. Surface OTUs, by contrast, were 0.84× as likely to be lost if they belonged to an individual’s hitting set, although more likely to be lost if they had a high indicator value.

One explanation for the preferential loss of identifying OTUs from skin is simply that they tend to be less abundant and are therefore less stable over time. While the positive correlation between abundance and stability would tend to support this, other factors suggest that this explanation is insufficient: the hazard model included terms for OTU abundance; indicator value was significantly positively correlated with abundance across all OTUs, individuals and seasons (Pearson’s *r* = 0.20, *p* < 0.05); and the hitting set algorithm prioritises abundant OTUs. It is more likely that identifying OTUs tend to be transients, acquired by an individual through chance environmental encounters to which other individuals were not exposed. Because these transient OTUs are less likely to be adapted to the skin environment and would not have their populations replenished by repeat exposure, they would be more exposed to eventual loss regardless of their abundance.

Abundant OTUs were also more likely to be deposited from skin to a household surface, with OTUs in the 99th percentile of abundance having 3.9× the baseline probability. More abundant OTUs have more cells available for deposition but also tend to persist for longer and thereby have increased time in which a chance deposition event can occur. Hitting set membership and lower indicator value slightly decreased the probability of a deposition event relative to the baseline, although the probability increased slightly with increasing indicator value with values between 0.9–1 having 1.6× baseline probability. This may reflect a slightly higher probability of a deposition event when an OTU is found on a higher proportion of an individual’s body sites, one of the properties measured by indicator value.

## Conclusions

This study suggests that the frequently invoked analogy between microbiota traces and fingerprints is misleading: unlike a fingerprint, skin microbiota changes over time both on the host and in microbial traces left on surfaces. Moreover, these changes are not random but select against low-abundance microorganisms as well as those most useful in identifying the individual who left the trace. Additional work is needed to better quantify the persistence of microbiota traces and how persistence depends on environmental factors. This study was only able to detect OTU loss on seasonal timescales; a similar study with a temporal resolution of hours or days could better quantify the expected lifespan of a trace. Further, this study examined only one type of indoor environment, a continually occupied residence where occupants were in frequent contact with household surfaces and input from other sources was comparatively unimportant. Forensic applications of microbiota matching may require the use of traces from other environments, such as public spaces or spaces with a significant microbial input from environmental sources, and for which data on matching accuracy is currently lacking.

## Methods

### Sample collection and DNA sequencing

Sample collection, genomic DNA (gDNA) extraction and sequencing were conducted as previously described [6]. Briefly, skin, surface and air samples were collected from a range of residences in Hong Kong across four seasons in 2014 (Table 1). Participants were instructed to maintain normal household routines, including cleaning schedules, during the study period. The V4 region of the 16S rRNA gene was amplified with the 515F/806R primer pair [20]. Library construction and paired-end sequencing were performed by the Health GeneTech Corporation (Taoyuan, Taiwan).

### OTU formation

Read quality control and OTU formation were performed following the UPARSE pipeline [21] using USEARCH version 8.1.1861. Raw sequencing reads were trimmed to a uniform length of 138 bp and filtered for a maximum of 1 expected error per read using the USEARCH command fastq_filter. These parameters were selected to maximise read retention while minimising expected errors, based on empirical analysis of the raw reads with the USEARCH command fastq_stats. Reads shorter than 138 bp or with ≥1 expected error after trimming were discarded. Forward and reverse reads were separated based on the identity of the first five nucleotides with the primer sequences, with sequences that did not perfectly match either primer in these positions discarded. As the paired ends did not overlap enough to allow reliable merging by alignment, only forward reads were used for subsequent analysis. Reads were dereplicated with a custom perl script, and dereplicated sequences were clustered at 97% sequence similarity with the USEARCH command cluster_OTUs to form OTU representative sequences, with singleton OTUs excluded. Probable chimeric OTU representative sequences were identified with the USEARCH command uchime_ref with the -strand plus option against the RDP classifier training database (downloaded 2 November 2015 from drive5.com/uchime/rdp_gold.fa). Reads were recruited to OTUs using the USEARCH command usearch_global with parameters -strand plus, -id 0.97, -maxaccepts 8, -maxrejects 64 and -top_hit_only. Consensus taxonomic lineages were assigned to OTUs with the QIIME [22] version 1.9.1 script assign_taxonomy.py against the Greengenes [23] version 13_8 97% similar 16S database. OTU representative sequences were aligned against the aligned Greengenes 97% similar 16S database with PyNAST [24] and the QIIME script align_seqs.py, and a tree constructed with FastTree [25] and the QIIME script make_phylogeny.py. With each batch of samples submitted to the sequencing facility, a kit control (i.e. DNA extracted from an unused swab or filter in parallel with samples [12]) was also submitted (total of nine kit control samples). Reads from these controls were not included in OTU formation, but control reads were subjected to the same quality control steps as sample reads and recruited against the OTU representative sequences generated from sample reads. A control OTU table was produced using a custom script that excluded OTUs matching any of the following conditions: representative sequence identified as chimeric; singleton OTU; representative sequence failed PyNAST alignment; OTU assigned to class ‘Chloroplast’ or family ‘mitochondria’. Following Flores et al. [26], any OTU found at ≥5% relative abundance in any control sample was designated a likely contaminant (Additional file 1: Figure S3). The relative abundance threshold (≥5%) was set higher than that used by Flores et al. (≥1%) due to the larger number of kit controls in this study. The final disposition of all reads sequenced for this project (including those removed for quality control purposes) is given in Additional file 1: Figure S4. A sample OTU table was produced that excluded OTUs matching any of the above conditions as well as likely contaminant OTUs (Additional file 2: Table S2). To account for differences in sequencing depth between samples, all samples were normalised by random subsampling to the number of reads in the most depauperate sample (1381 reads). To confirm that skin was the major source of surface OTUs, the Bayesian SourceTracker method [14] was used to estimate the proportional contributions of occupant skin and household air to surface samples within each household and season. Kit control samples were included as potential sources as a negative control.

### Microbiota matching

To confirm that microbiota comparison could be used to reliably match occupants to residences, and to investigate the effect of sampling delay on matching accuracy, SourceTracker was used to estimate the proportional contributions of occupant skin microbiota (‘sources’) to household surface microbiota (‘sinks’). Source estimation and scoring of match accuracy were performed as previously described [6]. Briefly, input OTU tables were prepared for all pairwise combinations of residence and season, with surface samples from the target residence in all seasons as sinks, and skin samples from occupants of all residences in the target season as potential sources. This arrangement made available all skin samples within each season as potential sources for each surface sample, without permitting sources to be drawn from multiple seasons. OTUs present in <10% of samples within each input table were excluded. Source contributions were estimated using the SourceTracker script sourcetracker_for_qiime.R (v1.0, downloaded 4 November 2015 from https://github.com/danknights/sourcetracker/archive/v1.0.tar.gz) with default settings. Each sink sample was considered to have an accurate match if the source contribution for occupants of that residence was greater than for occupants of any other residence (following Lax et al. [5]). The proportion of sink samples with accurate matches for each sampling delay (number of seasons between collection of skin and surface samples, expressed as an integer between −3 and +3) was taken as the accuracy rate for that delay. To investigate the effect of the number of possible matches on matching accuracy, microbiota matching with no season delay was repeated with separate random subsets of two, four, six and eight potential individuals or groups of individuals (including the correct match) selected as potential matches for each residence. Accuracy rates were calculated for each subset size as above. Weighted and unweighted UniFrac [15] distances were calculated between all samples using the QIIME script beta_diversity.py with default settings.

### OTU stability and deposition

OTU hitting sets [7] for each individual and body site were constructed to determine the stability of uniquely identifying OTUs and to explore the utility of hitting sets for microbiota matching. Hitting sets were generated with Franzosa et al.’s [7] python script idability.py (downloaded 13 December 2015 from https://bitbucket.org/biobakery/idability/get/default.tar.gz), run with the --meta_mode relab option to optimise for metagenome-like data in relative abundance format. The stability of winter hitting sets as unique identifiers across seasons was determined by running idability.py with the --codes flag, which attempts to re-identify individuals based on hitting sets.

Cox proportional hazard models [27] were used to determine the effect of a set of OTU properties on the stability of OTUs on occupant skin and household surfaces and on the deposition of OTUs from skin to surfaces. Proportional hazard models fit the instantaneous probability of an event (the ‘hazard’, in this case an OTU loss or deposition event) as a function of selected covariates, which can be either fixed or time-dependent. A useful property of these models is that as long as the baseline hazard function does not vary with time (i.e. the hazard varies only on some proportional combination of the covariates; the ‘proportional hazards assumption’), the proportional effect of each covariate on the hazard (the covariate’s ‘hazard ratio’) can be determined even if the baseline hazard is unknown. A second useful property is that these models allow for right-censored observations, where the period of observation may expire before an event is observed. These properties make proportional hazards models well suited for studying OTU stability and deposition over a fixed period.

Three models were created for this study. The first (the ‘skin stability’ models) examined the loss of skin OTUs as a function of time-dependent covariates representing OTU abundance, indicator value and membership in a hitting set for the modelled occupant body site. Each OTU on each body site served as a separate observation. Relative abundances were binned into four percentile ranges of increasing relative abundance (0–69th percentiles, 70–89th percentiles, 90–98th percentiles and 99th percentile) within each model (i.e. within each occupant body site). The OTU indicator value (IndVal [19]), a weighted measure of the OTU’s specificity (the proportion of the total abundance of that OTU that is found on the occupant) and fidelity (the proportion of body sites on that occupant in which the OTU is found), was calculated for each occupant in each season with the indval function of the R package labdsv [28] and binned into four ranges (0–0.2, 0.2–0.6, 0.6–0.9, 0.9–1). The hitting set membership covariate was a logical (true/false) value representing whether the OTU was a member of a hitting set for the modelled occupant and body site, in the season in which the OTU was first observed. The final covariate was the OTU’s indicator value (0–1; non-significant values masked to 0) for the modelled occupant. For each OTU, the observation period began in the season in which the OTU was first observed on the modelled occupant and body site and ended either in the last season in which it was observed (loss event) or in autumn (right-censoring with no loss event). If an OTU was not observed for an interim season(s) but then returned, this interim was not counted as a loss event.

The next set of models examined the stability of OTUs on household surfaces. Only OTUs found in at least one skin sample from an occupant of the modelled residence in any season as well as at least one surface sample from the modelled residence and surface in any season were included. This constrained the models to consider only OTUs that likely originated from occupant skin rather than other sources. Each OTU on each household surface served as a separate observation. Covariates were included for OTU abundance, indicator value (maximum value among occupants of that residence) and hitting set membership (of a hitting set for any occupant of that residence). Determination of loss events and right-censoring were as for the skin stability models.

The final model set investigated OTU deposition from skin to surface as a function of the same set of covariates used in the skin stability model. Each OTU on each body site served as a separate observation. The model covariates were set as with the skin stability models, and response variable was a deposition event, defined as an observation of the skin OTU on at least one household surface in the same or a later season as that of first observation on skin. OTUs present on a modelled surface before they were first observed on any occupants’ skin were excluded from the model.

All models were fit with the coxph function from the R library survival (version 2.38 [29, 30]) using survival objects built with the Surv function with right-censoring. Survival objects were constructed in the right-censored counting format, with each transition between seasons represented by an interval with associated covariate values for the season beginning that interval and a response variable representing whether a loss or deposition event occurred at the end of the interval. Significance for each covariate was determined as the two-tailed *p* < 0.05 of the observed Wald statistic (*z*, ratio of the fitted coefficient to its standard error) under the null hypothesis of a hazard ratio of 1 (i.e. no effect), with non-significant covariates rejected.

## Additional files

Additional file 1: Table S1. Taxonomy of hitting set OTUs. Each assignment of an OTU to an individual’s hitting set is counted once (‘Count’); OTUs assigned to more than one hitting set are counted more than once. ‘Source’ indicates families listed as indicative of a particular source environment, following the scheme of Dunn et al. [9]. **Figure S1.** Microbiome matching accuracy as a function of the number of potential matches. For each household for which matching was performed, random subsets of two, four, six and eight potential matches (individuals or groups of individuals) were randomly selected for SourceTracker-based microbial matching. Accuracy was determined as the proportion of residences for which SourceTracker estimated the correct set of occupants’ skin microbiota as the major source for the residences’ surface microbiota. **Figure S2.** Accuracy of winter hitting sets in identifying individuals in later seasons. Sets that identified both the correct individual and one or more additional individuals were classified as ‘true positive + false positive’; sets that did not identify the correct individual but did identify one or more other individuals were classified as ‘false negative + false positive’. **Figure S3.** OTUs identified as likely contaminants, based on their abundance in the negative control (blank) samples. Taxonomy is given to the binomial level where available. **Figure S4.** Final disposition of all raw reads sequenced for this project. Reads were assigned to an OTU and included in the analysis (‘Assigned OTU’), or excluded for one of the following reasons: failed to be clustered in a non-singleton OTU (‘No OTU assignment’), likely chimeric (‘Chimera’), assigned to OTU found in high abundance in negative controls (‘Contaminant’), assigned to OTUs with non-bacterial phylogeny (‘Chloroplast’ or ‘Mitochondria’), assigned to OTU that could not be aligned for phylogenetic tree construction (‘Failed alignment’), failed initial read quality control (‘Failed QC’), unused mate pair (‘Reverse’), unable to be unambiguously identified as a forward or reverse read (‘Unsorted’) or belonged to a negative control sample (‘BlankForward’, ‘BlankReverse’ or ‘BlankUnsorted‘). (PDF 123 kb)
Additional file 2: Table S2. OTU table. Final OTU table generated from the sequencing data, following all quality control steps. ‘Count’ gives the number of reads for a given OTU identified in a given sample. For skin samples, indicator value, indicator *p* value, and whether the OTU was a member of a hitting set for that individual in that season are indicated. ‘NA’ values indicate properties not relevant to a given sample (e.g. air samples are not associated with an individual). (TSV 30906 kb)
