# Supplementary material for: Microbiota fingerprints lose individually identifying features over time
Source: Microbiome. 2017 Jan 9;5:1. doi: 10.1186/s40168-016-0209-7 (PMC5234115; doi:10.1186/s40168-016-0209-7)

Microbiota fingerprints lose individually identifying features over time

Supplementary Information

David Wilkins, Marcus H.Y. Leung, Patrick K.H. Lee

Table S1: Taxonomy of hitting set OTUs. Each assignment of an OTU to an individual's hitting set is counted once ('Count'); OTUs assigned to more than one hitting set are counted more than once. 'Source' indicates families listed as indicative of a particular source environment, following the scheme of Dunn *et al.* (2013).

Rank	Family	Source	Count
1	*Moraxellaceae*		302
2	*Sphingomonadaceae*		185
3	*Unknown*		166
4	*Rhodobacteraceae*		159
5	*Weeksellaceae*		119
6	*Enterobacteriaceae*	Leaf	100
7	*Deinococcaceae*	Leaf	95
8	*Xanthomonadaceae*		93
9	*Corynebacteriaceae*	Human skin	88
10	*Micrococcaceae*		77
11	*Pseudomonadaceae*		68
12	*Tissierellaceae*		51
13	*Methylobacteriaceae*		49
14	*Bacillaceae*		47
15	*Microbacteriaceae*		47
16	*Gordoniaceae*		45
17	*Comamonadaceae*		43
18	*Oxalobacteraceae*		41
19	*Ruminococcaceae*	Human stool	35
20	*Dermatophilaceae*		34
21	*Actinomycetaceae*	Human oral cavity	33
22	*Neisseriaceae*	Human oral cavity	33
23	*Caulobacteraceae*		32
24	*Rhodospirillaceae*		32
25	*Chitinophagaceae*		28
26	*Acetobacteraceae*		27
27	*Prevotellaceae*	Human oral cavity	27
28	*Streptococcaceae*		26
29	*Staphylococcaceae*	Human skin	24
30	*Brevibacteriaceae*		23
31	*Pseudonocardiaceae*		23
32	*Dietziaceae*		22
33	*Aerococcaceae*		20
34	*Porphyromonadaceae*		20
35	*Nocardiaceae*		19
36	*Sphingobacteriaceae*		19
37	*Cytophagaceae*		18
38	*Flavobacteriaceae*		18
39	*Bacteroidaceae*	Human stool	16
40	*Mycobacteriaceae*		16
41	*Pasteurellaceae*	Human oral cavity	16
42	*Propionibacteriaceae*	Human skin	16
43	*Paraprevotellaceae*		15
44	*Intrasporangiaceae*		15

Rank	Family	Source	Count
45	*Veillonellaceae*	Human oral cavity	14
46	*Lachnospiraceae*	Human stool	13
47	*Rhizobiaceae*		12
48	*Rhodocyclaceae*		12
49	*Xenococcaceae*		12
50	*Leuconostocaceae*		11
51	*Bifidobacteriaceae*		10
52	*Carnobacteriaceae*		10
53	*Dermacoccaceae*		10
54	*Leptotrichiaceae*	Human oral cavity	10
55	*Burkholderiaceae*		9
56	*Lactobacillaceae*		9
57	*Exiguobacteraceae*		7
58	*Beijerinckiaceae*		7
59	*Brucellaceae*		7
60	*Nocardioidaceae*		7
61	Ellin6075		6
62	*Halomonadaceae*		6
63	*Planococcaceae*		6
64	*Dermabacteraceae*		5
65	*Fusobacteriaceae*	Human oral cavity	5
66	*Gemellaceae*		5
67	*Patulibacteraceae*		5
68	*Psychromonadaceae*		5
69	*Chthoniobacteraceae*		4
70	*Alteromonadaceae*		4
71	*Anaerolinaceae*		4
72	*Bacteriovoracaceae*		4
73	*Enterococcaceae*		4
74	*Erythrobacteraceae*		4
75	*Paenibacillaceae*		4
76	*Rikenellaceae*	Human stool	3
77	*Thermotogaceae*		3
78	*Vibrionaceae*		3
79	*Williamsiaceae*		3
80	*Aeromonadaceae*		2
81	*Bartonellaceae*		2
82	*Bdellovibrionaceae*		2
83	*Clostridiaceae*		2
84	*Coriobacteriaceae*		2
85	*Gemmataceae*		2
86	*Haliangiaceae*		2
87	*Planctomycetaceae*		2
88	*Polyangiaceae*		2
89	*Rhodothermaceae*		2
90	*Thermaceae*		2
91	*Chromatiaceae*		1

Rank	Family	Source	Count
92	*Cloacamonaceae*		1
93	*Mogibacteriaceae*		1
94	*Blattabacteriaceae*		1
95	*Bradyrhizobiaceae*	Soil	1
96	*Cryomorphaceae*		1
97	*Geodermatophilaceae*		1
98	*Hyphomicrobiaceae*	Soil	1
99	*Methanobacteriaceae*		1
100	*Methanosaetaceae*		1
101	*Nakamurellaceae*		1
102	*Phormidiaceae*		1
103	*Pirellulaceae*		1
104	RFP12		1
105	SHA-116		1
106	*Spirochaetaceae*		1
107	*Streptomycetaceae*		1

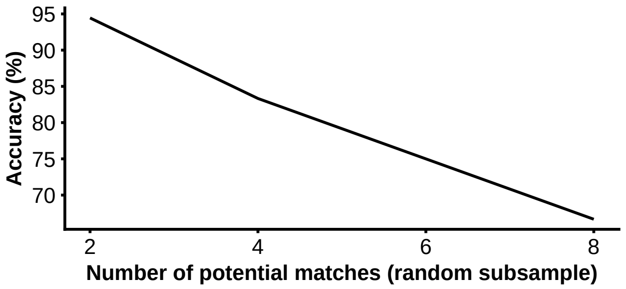

Figure S1: Microbiome matching accuracy as a function of the number of potential matches. For each household for which matching was performed, random subsets of two, four, six and eight potential matches (individuals or groups of individuals) was randomly selected for SourceTracker-based microbial matching. Accuracy was determined as the proportion of residences for which SourceTracker estimated the correct set of occupants' skin microbiota as the major source for the residences' surface microbiota.

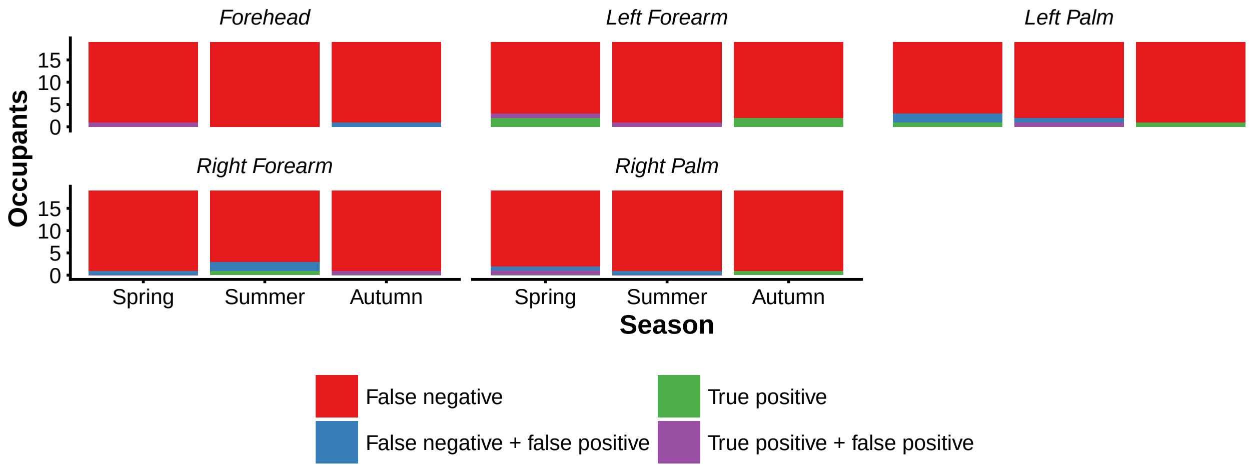

Figure S2: Accuracy of winter hitting sets in identifying individuals in later seasons. Sets that identified both the correct individual and one or more additional individuals were classified as 'true positive + false positive'; sets that did not identify the correct individual but did identify one or more other individuals were classified as 'false negative + false positive'.

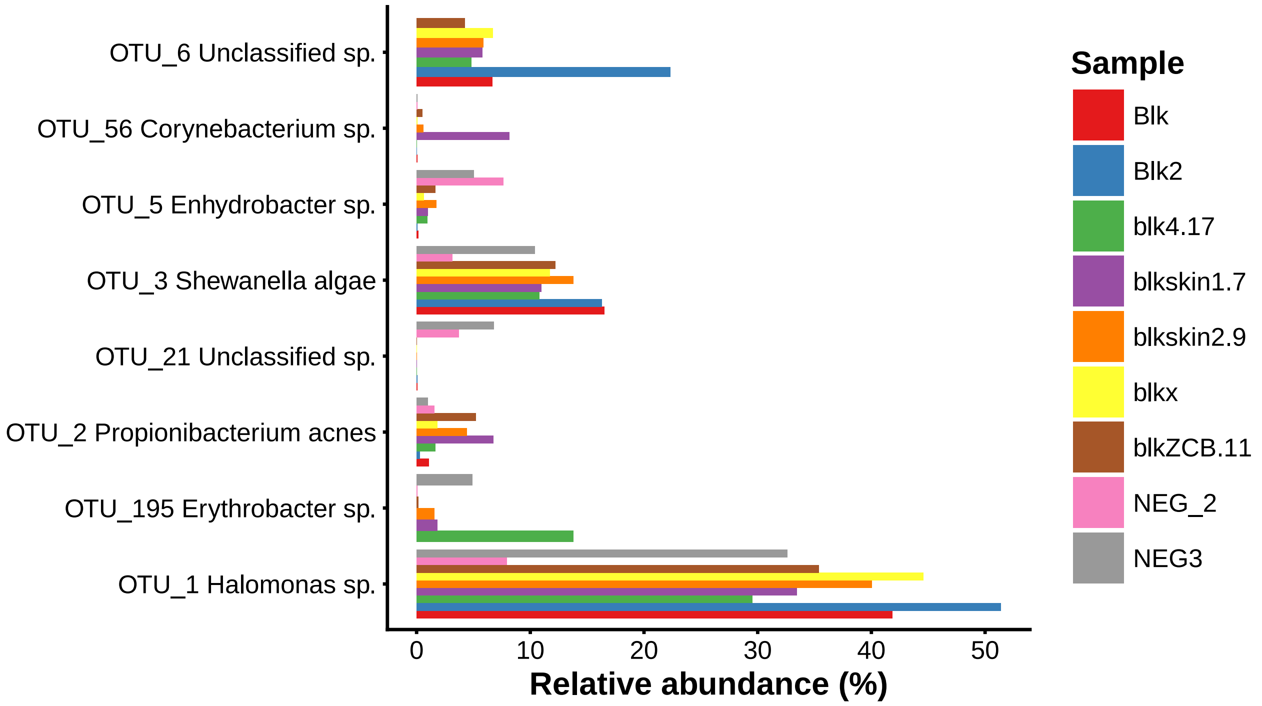

Figure S3: OTUs identified as likely contaminants, based on their abundance in the negative control (blank) samples. Taxonomy is given to the binomial level where available.

Figure S4 (following page): Final disposition of all raw reads sequenced for this project. Reads were assigned to an OTU and included in the analysis ('Assigned OTU'), or excluded for one of the following reasons: failed to be clustered in a non-singleton OTU ('No OTU assignment'), likely chimeric ('Chimera'), assigned to OTU found in high abundance in negative controls ('Contaminant'), assigned to OTUs with non-bacterial phylogeny ('Chloroplast' or 'Mitochondria'), assigned to OTU that could not be aligned for phylogenetic tree construction ('Failed alignment'), failed initial read quality control ('Failed QC'), unused mate pair ('Reverse'), unable to be unambiguously identified as a forward or reverse read ('Unsorted'), or belonged to a negative control sample ('BlankForward', 'BlankReverse' or 'BlankUnsorted').

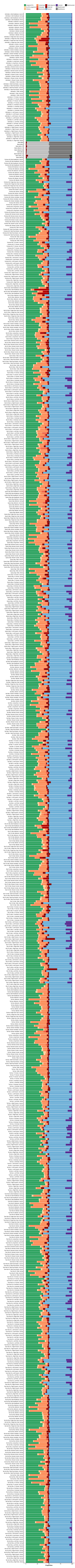

Supplement: Additional file 1: Table S1. — Taxonomy of hitting set OTUs. Each assignment of an OTU to an individual’s hitting set is counted once (‘Count’); OTUs assigned to more than one hitting set are counted more than once. ‘Source’ indicates families listed as indicative of a particular source environment, following the scheme of Dunn et al. [9]. Figure S1. Microbiome matching accuracy as a function of the number of potential matches. For each household for which matching was performed, random subsets of two, four, six and eight potential matches (individuals or groups of individuals) were randomly selected for SourceTracker-based microbial matching. Accuracy was determined as the proportion of residences for which SourceTracker estimated the correct set of occupants’ skin microbiota as the major source for the residences’ surface microbiota. Figure S2. Accuracy of winter hitting sets in identifying individuals in later seasons. Sets that identified both the correct individual and one or more additional individuals were classified as ‘true positive + false positive’; sets that did not identify the correct individual but did identify one or more other individuals were classified as ‘false negative + false positive’. Figure S3. OTUs identified as likely contaminants, based on their abundance in the negative control (blank) samples. Taxonomy is given to the binomial level where available. Figure S4. Final disposition of all raw reads sequenced for this project. Reads were assigned to an OTU and included in the analysis (‘Assigned OTU’), or excluded for one of the following reasons: failed to be clustered in a non-singleton OTU (‘No OTU assignment’), likely chimeric (‘Chimera’), assigned to OTU found in high abundance in negative controls (‘Contaminant’), assigned to OTUs with non-bacterial phylogeny (‘Chloroplast’ or ‘Mitochondria’), assigned to OTU that could not be aligned for phylogenetic tree construction (‘Failed alignment’), failed initial read quality control (‘Failed QC’), unused mate pair (‘Revers [file 40168_2016_209_MOESM1_ESM.pdf]
